# Annotations

**Published:** 1911-01-14

**Authors:** 


### ANNOTATIONS.

### Mark Twain's Appetite Cure.

On the subject of hunger Mark Twain has one of his best short stories, "The Appetite Cure," wherein the sensations of the overfed Londoner, locked in a room, in the bracing air of the Alps, reading literature that deals with shipwrecked sailors, entombed miners, and such-like hungry persons, are delightfully described. The hero (if hero he can be called) of this extravaganza has to stay without food until he can bring himself to start his meal with an omelette of rotten eggs. The moral of the story is that when feeling unwell the best thing to do is to drop a meal, and get really hungry, when perfect health will have returned. There is certainly much that is very sound in this advice, and in many cases it will cure. On the other hand, it would be dangerous to give it indiscriminately. The determining factor of the onset of many acute infective disorders is probably a temporary lowering of the resistance, and a long period without food may often be one of the causes of this. On the other hand, the usefulness of a periodical fast is now generally acknowledged by diet-specialists, and it has even been hinted that the improved racial stamina of the Jews and Mohammedans is due, in a measure at least, to their insistence on dietetic rules in which fasting, in some form or other, plays a prominent part.

### A Good Word for Tobacco.

In the opinion of Cavallaro, who writes in *La Stomatologia*, Milan, tobacco has a very great bactericidal power depending to some extent on its content in nicotine and partly on the resistance of bacteria present. In all cases tobacco sterilises the saliva and does no damage to the teeth, which become and remain black if not properly looked after. A small quantity of nicotine stimulates salivary excretion, but a large amount diminishes it. It is wrong to attribute inflammations of the gums and buccal mucous membrane to the influence of tobacco. This last is only the determining factor in a pre-existing inflammatory process, whether latent or manifest. It is, moreover, by no means proved that epithelioma of the tongue and lips is exclusively due to tobacco smoking. The author, relying on this antiseptic action of tobacco, calls for an increase, and not a diminution, in the ranks of smokers, an invitation with which the hardened slaves of "my lady nicotine" will not be disposed to quarrel.

### The Night Bell.

Medical men who are called out of bed at dawn (and that is not very early at this time of year) have some consolation for the disturbance of their rest in seeing the most lovely time of day. It is true we appreciate this more in retrospect than we do at the time. But an early morning call has its advantages from an artistic point of view. The air is always softest then, the sky shows its most alluring hues, and the leafless branches of the trees, standing out against these variegated backgrounds of tints, give patterns and designs that all man's ingenuity cannot rival. If a poll of practitioners were taken we think it would be found that the majority prefer this time to all others in which to be disturbed, while it is probable that the referendum would substantiate the theory that the worst time for a night call is during that period of profound sleep just after going to bed. Worst of all undoubtedly is to be called out just after getting into bed and before sleep has actually come. The psychology of the situation deserves some attention. There is scope for an essay on "The Doctor's Night Call."

### Free Antitoxin.

The provisions of the Order with respect to diphtheria antitoxin published by the Local Government Board in August 1910 have been taken advantage of by many local authorities. As an example of regulations which may almost be termed model, the arrangements made by the Council of the Royal borough of Kensington are worthy of citation. In a circular by the Medical Officer of Health to all practitioners in the district, it is intimated that diphtheria antitoxin will henceforth be supplied free of charge to them for the treatment and prevention of diphtheria among the poor. A supply of this substance is kept for such gratuitous distribution at the Town Hall and by three reputable chemists carrying on business in various parts of this large borough. A fee of five shillings will be paid to practitioners by the Council for the administration of antitoxin to poor persons; and the Council's serum is, naturally, only to be used for those who cannot afford to pay for it themselves. To ensure the observance of the latter regulation practitioners must sign a book every time they apply for serum, stating for whom it is required. The dosage is left to the discretion of the doctor, but the Council suggest that 2,000 units is the most that is required for prophylaxis, and that 8,000 is the least that is likely to be of service in a really severe case. The circular concludes with statistics of the mortality from diphtheria before and since the introduction of antitoxin, and with certain conclusions which it is important to have generally accepted by the profession. One of these is that isolation of contacts is still of the first importance, prophylactic serum treatment standing second to it. Another is that antitoxin is definitely proved to have caused death in healthy persons in very rare and exceptional cases. Another that whenever signs of laryngeal stridor cannot be met by clear proof of the absence of diphtheria, antitoxin should be administered forthwith; and a fourth that in doubtful cases specific treatment should be deferred until after a bacteriological examination or the appearance of undoubted membrane. Compliance with the antitoxin order has thus been secured, in Kensington at least, on terms which provide equally for the safety of the public, the restriction of diphtheria, and the due recognition of the labour of the medical profession in securing both these objects.

